# From ADAS to Material-Informed Inspection: Review of Hyperspectral Imaging Applications on Mobile Ground Robots

**DOI:** 10.3390/s25082346

**Published:** 2025-04-08

**Authors:** Daniil Valme, Anton Rassõlkin, Dhanushka C. Liyanage

**Affiliations:** 1 Department of Electrical Power Engineering and Mechatronics, Tallinn University of Technology, 19086 Tallinn, Estonia; 2 Kuressaare College, Tallinn University of Technology, 93811 Kuressaare, Estonia; dhanushka.liyanage@taltech.ee

**Keywords:** hyperspectral imaging (HSI), Advanced Driver Assistance Systems (ADAS), navigation, inspection, mobile ground robots

## Abstract

Hyperspectral imaging (HSI) has evolved from its origins in space missions to become a promising sensing technology for mobile ground robots, offering unique capabilities in material identification and scene understanding. This review examines the integration and applications of HSI systems in ground-based mobile platforms, with emphasis on outdoor implementations. The analysis covers recent developments in two main application domains: autonomous navigation and inspection tasks. In navigation, the review explores HSI applications in Advanced Driver Assistance Systems (ADAS) and off-road scenarios, examining how spectral information enhances environmental perception and decision making. For inspection applications, the investigation covers HSI deployment in search and rescue operations, mining exploration, and infrastructure monitoring. The review addresses key technical aspects including sensor types, acquisition modes, and platform integration challenges, particularly focusing on environmental factors affecting outdoor HSI deployment. Additionally, it analyzes available datasets and annotation approaches, highlighting their significance for developing robust classification algorithms. While recent advances in sensor design and processing capabilities have expanded HSI applications, challenges remain in real-time processing, environmental robustness, and system cost. The review concludes with a discussion of future research directions and opportunities for advancing HSI technology in mobile robotics applications.

## 1. Introduction

The term hyperspectral imaging (HSI) in remote sensing emerged in the late 1980s to describe the results obtained through imaging spectrometry. These advancements were achieved at the Jet Propulsion Laboratory (JPL) of the California Institute of Technology in Pasadena, in collaboration with the National Aeronautics and Space Administration (NASA) [1]. Hyperspectral images, also called hypercubes, consist of hundreds of images captured at different wavelengths stacked together. Each pixel contains information across the electromagnetic spectrum, producing a spectral signature that can be used for material identification.

While conventional imaging systems like RGB (red–green–blue) cameras capture data in three broad spectral bands, HSI systems collect information across hundreds of narrow, contiguous bands. This high spectral resolution is crucial for real-world applications where materials exist in complex mixtures, and environmental factors affect measurements. The rich spectral information enables the separation and identification of distinct material components, even when they appear visually similar in conventional imaging. The evolution of HSI technology has been driven by three parallel developments: advances in sensor design, sophisticated data processing algorithms, and increased computational power [2]. The advancement of HSI sensor design, computational power, and data processing algorithms has extended its applications from aerospace to diverse fields, including medicine [3], agriculture [4], waste management [5], environmental monitoring [6], forestry [7], food quality assessment [8], cultural heritage preservation [9], and security [10].

While the application of HSI has been extensively discussed in aerial [11,12], spaceborne [13,14], and indoor studies [5], its integration with mobile ground robots—such as autonomous vehicles, quadruped robots, and unmanned ground vehicles (UGVs)—has received comparatively less attention. Existing reviews primary focus on the integration of HSI for precision agriculture purposes [4,15,16]. In mobile robotics and relevant applications, HSI sensors are primarily deployed for two key tasks:

- Navigation: assisting autonomous vehicles and UGVs in environment perception, terrain classification, and road condition analysis.
- Inspection and Monitoring: supporting non-destructive material analysis for various purposes.

For both tasks, HSI can provide a new layer of information describing the materials of the objects surrounding the platform. In the case of autonomous driving and ADAS systems, vehicles must adapt to drastically different scenarios influenced by the environment type, weather conditions, presence of objects, time of day, and vehicle speed. In this context, HSI provides valuable input for vehicle control, enhancing scene understanding beyond the capabilities of traditional RGB-based image processing. HSI can predict road conditions and distinguish objects based on spectral properties, particularly in off-road and rural environments with limited infrastructure.

Similarly, HSI sensors mounted on mobile platforms for inspection and monitoring can provide valuable spectral insights for a range of applications. Combined with the mobility of robotic platforms, non-destructive HSI has the potential to become another tool in asset management and predictive maintenance, particularly in hard-to-reach environments such as wind farms. In the context of mining applications, HSI can contribute to intelligent mineral mapping and tunnel inspection, enabling automated material classification and improving safety and efficiency in underground operations.

The review aims to investigate and analyze the existing studies related to the application of HSI on board of different mobile platforms and provide an overview on integration related aspects. The study discusses the navigation and inspection separately, as the end goal of HSI application is different. In addition, the review includes the relevant studies, where the experiments were performed using a controlled lab setup or outdoors using the handheld devices. These studies indicate the applicability of the HSI for specific tasks but might require in-field testing with mobile platforms.

The paper is structured as follows. Section 2 provides an overview of the technological advancements enabling HSI implementation in mobile systems, including the deployment of HSI on mobile platforms, and examines the key factors influencing the integration of HSI sensors with platforms. Section 3 reviews the main hardware components of the HSI system and discusses the integration architecture in general. Section 4 summarizes the applicability of HSI acquisition modes for navigation and inspection purposes. The first part of Section 5 reviews the existing research on the application of spectral imaging on board of mobile platforms for ADAS systems and unstructured terrain scenarios, alongside the application-related details, hardware used, dataset annotation approaches, classification methods, and common challenges. The second part is focused on applications related to inspection tasks. Section 6 explores two critical factors affecting optical systems: variation in illumination and contamination of optical components. Section 7 discusses the integration of HSI in mobile platforms for navigation and inspection tasks, with the key findings summarized in the SWOT table.

## 2. HSI for Mobile Platforms

HSI combines spectroscopy and digital imaging, enabling both spatial and spectral information collection from an object. The modifier “hyper” highlights the ability of the sensor to capture the data in a few hundred narrow, contiguous bands and depending on the application, the sensing range may include visible light (VIS, 0.38–0.75 µm), near-infrared (NIR, 0.7–1.0 µm), short-wave infrared (SWIR, 1.0–2.5 µm), mid-infrared (MWIR, 3–5 µm), and long-wave infrared (LWIR, 8–12 µm) parts of the electromagnetic spectrum. These sensors quantify the radiant flux intensity for a specific surface area and wavelength, typically measured in units of W/sr·m^2^ (watts per steradian per square meter) [17].

**Figure 1:**
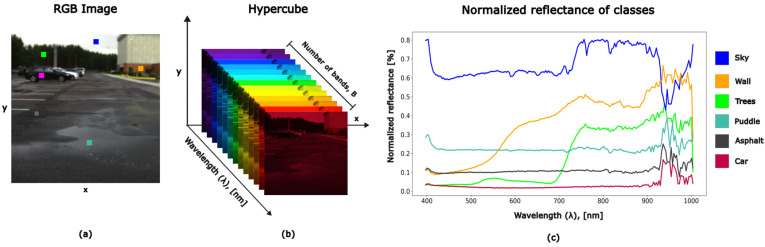
Example of a scene acquired using the Specim IQ hyperspectral camera in the parking area of Tallinn University of Technology: (**a**) RGB image of the scene with color-coded markers indicating selected material classes; (**b**) corresponding HIS cube, representing spatial dimensions (x, y) and spectral dimension (λ); (**c**) normalized reflectance (spectral signatures) of different materials common for urban environments, including sky, walls, trees, puddles, asphalt, and cars. The spectral signatures, extracted from the corresponding spatial coordinates, demonstrate the reflectance characteristics of each material class across the visible and near-infrared spectrum.

In contrast, conventional RGB camera sensors acquire three values corresponding to three bands of the VIS [18]. In the case of multispectral imaging (MSI), the same scene is acquired using only several wavebands [19]. Hyperspectral sensors typically produce data in the form of hyperspectral images or three-dimensional (3D) data cubes structured as (x, y, λ), which have B spectral bands and x × y pixels [17], as shown in Figure 1. Hundreds of spectral values are not crucial to material identification. But in real-world situations, materials usually exist in mixes, and ambient factors might affect measurements. As a result, a greater number of spectral bands is required to properly separate and describe the distinct material components [2].

### 2.1. Sensing Modes

**Figure 2:**
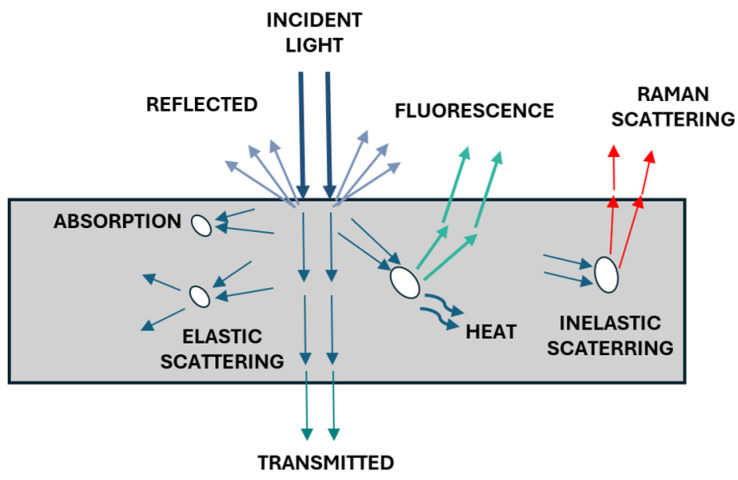
Light–matter interactions, showing reflection, transmission, fluorescence, and Raman scattering. Fluorescence and Raman scattering have different wavelengths, indicated by colored arrows [21].

Spectral imaging utilizes the fundamental principles of spectroscopy, enabling remote material identification through the analysis of light–matter interactions [20], including absorption, transmission, reflection, and scattering processes, as presented in Figure 2. The incident light can be reflected back after absorption and multiple scattering events or thorough energy transfer and particles in the material, resulting in fluorescence of Raman scattering, typically at longer wavelengths [21]. Depending on the type of interaction, the analysis is performed in different sensing and acquisition modes. The selection of both modes correlates with the sample size as well as the acquisition time available to perform analysis [22]. The sensing modes, sometimes referred to as measurement modes, include reflectance, transmittance, interacting, transflectance, fluorescence, and Raman.

**Figure 3:**
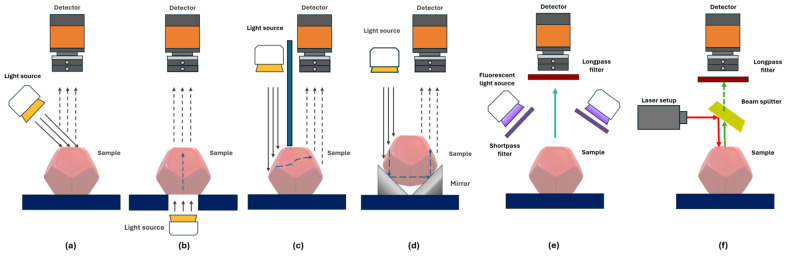
Sensing modes (**a**) reflectance, (**b**) transmittance, (**c**) interactance, (**d**) transflectance, (**e**) fluorescence, and (**f**) Raman.

In reflectance mode (Figure 3a), the detector acquires the light reflected from the sample surface. The mode is used to provide information about physical (size, shape, and surface texture) and material composition. This mode is widely used in various fields, including remote sensing. Next, the transmittance mode (Figure 3b) captures the light passing through the material, where the internal structure must be examined [23,24]. It is applicable with liquids, vapors, and homogenized and solid opaque samples [25,26]. The primary challenge in transmittance mode is the low signal levels caused by light attenuation [8], necessitating the use of high-intensity light sources and highly sensitive detectors [27].

Interactance mode (Figure 3c) represents a hybrid approach, combining elements of both reflectance and transmittance modes [21]. In this configuration, the light source and imaging area are physically separated by a light barrier, enabling the simultaneous analysis of surface characteristics and internal sample properties [25]. In transflectance mode, the light passes the sample twice (Figure 3d). This is achieved by placing a mirror on the opposite side from the light source, which reflects the light back through the sample.

Fluorescence mode (Figure 3e) measures light emitted by materials after energy absorption at a different (typically longer) wavelength than the excitation light [22]. It is used in biomedical applications for tissue identification [28]. Raman mode (Figure 3f) utilizes monochromatic laser sources, typically operating in the visible or near-infrared range (785 and 830 nm). This mode generates spectral information based on the inelastic scattering of photons [22,29], providing detailed molecular composition analysis of the sample. Both Raman and fluorescence demand an intense excitation light source and a high-performance detector to maintain sufficient signal quality [25].

Reflectance mode dominates in in-field measurements due to its practicality. Since reflectance is an inherent material property independent of external conditions, it serves as a strong discriminator for classification [17]. In mobile applications, measurements can be taken without physical contact with samples and with minimal constraints on the distance between the sensor and the sample. An important consideration for mobile robots is their ability to utilize both natural and artificial light sources. The raw data produced by a sensor are digital numbers (DNs), values sometimes referred as digital counts, which correspond to the incident flux on the detector at specific wavelengths [30,31]. Afterward, DNs are converted into two key measurements: radiance and reflectance. Reflectance is calculated as the ratio of incident light energy to reflected energy at each wavelength. Unlike radiance, reflectance is considered an intrinsic material property, largely independent of external factors such illumination intensity or direction [17,32].

Raman spectroscopy enables precise chemical analysis crucial for applications like material detection [33]. Commercial Raman sensors typically have a limited inspection area. Despite that, these systems achieve highly specific molecular identification capabilities, making them valuable for the targeted analysis of small samples in field conditions. Other modes require a more sophisticated setup to control the acquisition process, which can be achieved in the laboratory environments [34,35]. However, these measurements could potentially be conducted in the field by transforming mobile platforms into mobile laboratories equipped with manipulators for sample handling and dedicated measurement chambers like the ones discussed in [36].

### 2.2. Parameters of the HSI System

**Figure 4:**
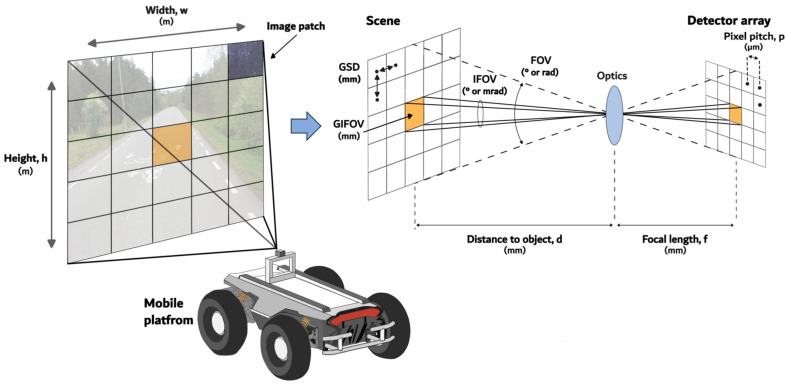
HSI system mounted on a mobile platform. Field of View (FOV) determines the total angular coverage, instantaneous field of view (IFOV) represents the angular resolution per pixel, and ground instantaneous field of View (GIFOV) translates this into real-world distances. Ground sampling distance (GSD) quantifies the spacing between adjacent pixels on the observed surface, while focal length (f) and pixel pitch (p) influence image resolution.

The integration of the HSI into a mobile platform requires a detailed understanding of dedicated parameters. These parameters influence both the applicability of the system for specific tasks and the quality of the data obtained. These parameters are illustrated in Figure 4.

Spatial resolution refers to the capability of the sensor to distinguish and separately identify closely situated objects [37]. It is defined by the design of the sensor and the distance between the sensor and the object of observation. Each detector of the array (pixel) measures the energy coming from a specific patch of the scene. The patch size has an inverse relationship with spatial resolution. Smaller patches provide more detailed spatial information from the image [38].

The field of view (FOV) is a fundamental parameter that defines the total angular coverage captured by a sensor in a single image. FOV is primarily determined by the optical system’s focal length (f) and the physical dimensions of the detector array. The optical system focuses the incoming radiation onto the detector array, where each pixel (defined by its pitch p) samples a specific portion of the scene. In remote sensing, spatial resolution is commonly characterized by the instantaneous field of view (IFOV), which represents the angle subtended by the geometrical projection of a single detector pixel [39,40]. IFOV is measured in milliradians (mrad) and corresponds to the smallest detectable object in the image.

While IFOV provides an angular measurement, Ground IFOV (GIFOV) converts this to practical ground distances by projecting the IFOV onto the area of inspection, providing measurements in meters that are more directly useful for analysis [41]. The ground sample distance (GSD) represents the spatial resolution metric defined as the linear distance between adjacent pixel centers as projected onto the terrain surface [42]. This measurement quantifies the ground-level spacing between consecutive image elements, effectively characterizing the spatial granularity of remotely sensed data. The larger the GSD, the lower the spatial resolution.

**Figure 5:**
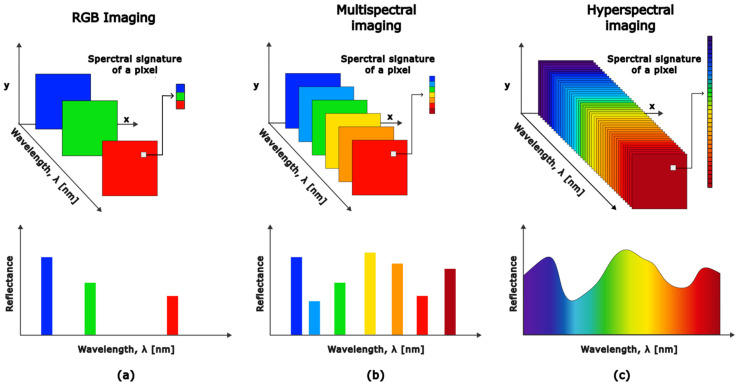
Comparison of spectral resolution of imaging techniques: (**a**) RGB imaging captures three broad spectral bands (red, green, and blue), limiting spectral resolution; (**b**) multispectral imaging collects several discrete spectral bands, providing more spectral detail; (**c**) hyperspectral imaging acquires a continuous spectrum with numerous narrow bands, allowing for precise analysis.

Spectral resolution is defined as the range of the electromagnetic spectrum and the number of spectral bands measured by the sensor. An imaging sensor can be responsive to a wide range of the electromagnetic spectrum but still have low spectral resolution if it captures only a few spectral bands within that range. Conversely, a sensor that operates within a narrow range of the electromagnetic spectrum but acquires a large number of spectral bands will have high spectral resolution, as it can distinguish between scenes with similar or closely related spectral signatures [38]. HSI achieves high spectral resolution by capturing hundreds of spectral measurements per pixel, enabling detailed spectral differentiation. In contrast, (MSI) typically operates with significantly fewer bands, usually fewer than 10, though some systems utilize dozens [8]. The fine spectral resolution of the HSI systems enables more precise differentiation of materials based on the continuous signatures. The spectral resolution associated with RGB, MSI, and HSI imaging techniques is illustrated in Figure 5.

Lastly, temporal resolution describes how frequently a sensor takes pictures of the same spot on Earth. The time interval between successive observations at the same location on Earth’s surface is basically known as the revisit time [43]. Geometric accuracy metrics measure how accurately spatial relationships between objects in a real-world scene are preserved and represented in the resulting image. It is crucial in applications where the accurate position of the objects within the scene is important [44].

### 2.3. HSI Deployment on Mobile Platform

**Figure 6:**
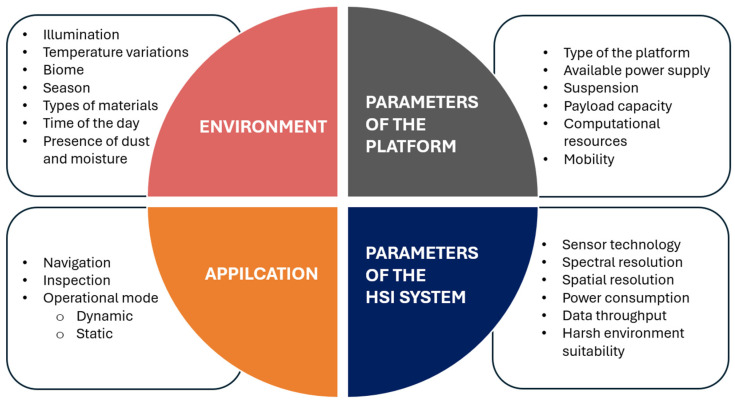
Factors influencing integration of HSI sensor with mobile platform.

Deployment of the HSI sensor on the platform is a task requiring careful consideration of multiple interrelated groups of factors, as summarized in Figure 6. The first group of factors is presented by the environment where the platform operates. These include specific biome characteristics that determine the types of materials encountered as well as the presence of moisture and dust potentially interfering with the measurements. On a high level, spectral signatures characterizing the same object may vary depending on the season due to associated biological processes like chlorophyll degradation. On the low level, the low-level signatures may be influenced by the moisture on the surface of the objects caused by the changes in the temperature throughout the day. Lastly, the illumination of the scene plays a crucial role in HSI integration, as the quality and stability of illumination within the environment directly affects the quality of acquired spectral data. The variations in illumination conditions can occur due to the time of day, weather conditions, or shadowing effects, requiring careful consideration in system design and data processing algorithms to ensure reliable spectral measurements.

The second group of factors is dedicated to the application of the HSI system. In the case of the application of spectral imaging for navigation purposes, the scene must be acquired at the highest possible speed to provide information about the environment for decision making. In contrast, inspection tasks using mobile platforms typically operate at lower acquisition speeds, allowing for more detailed spectral data collection. The system design depends heavily on whether it operates in dynamic mode during movement or static mode while stationary. Dynamic operation during navigation requires robust motion compensation and fast data processing, while static operation during inspection enables higher-precision measurements and more thorough spectral analysis. These application-specific requirements directly impact the selection of sensor parameters, processing algorithms, and overall system architecture.

The factors of the third group are related to the parameters of the HSI system, which are primarily defined by the parameters of the sensor used in the setup. Spectral resolution must be sufficient for the intended application, enabling accurate material discrimination. Spatial resolution defines the ability to detect and distinguish features of interest at various distances. Both resolutions contribute to the data throughput, which is important in terms of processing resources.

Lastly, the fourth group of factors relates to the parameters of the mobile platform. The payload capacity limits must be carefully evaluated to ensure the platform can accommodate components of the HSI system, like stabilizers or light sources. Despite the payload, the power consumption of the system associated with both acquisition and processing should be considered. The available power supply of the platform defines both operational duration and processing capabilities—whether computations are performed in real-time onboard or postponed for offline analysis. Additionally, the suspension system of the mobile platform mitigates motion-induced artifacts in HSI images by reducing camera instability during data acquisition.

## 3. Components of the HSI System

**Figure 7:**
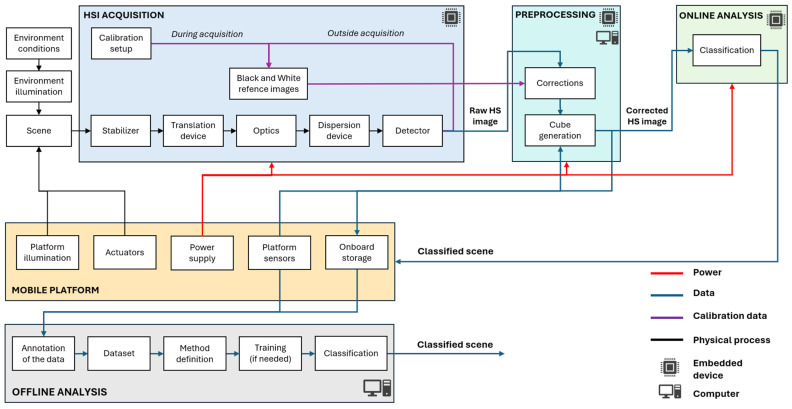
Schematic diagram. HSI integration on a mobile platform, covering acquisition, preprocessing, and classification stages for online and offline processing scenarios.

The parameters of the HSI system are defined by the parameters of its components. The traditional HSI laboratory setup usually has the following parts: an objective lens, a wavelength dispersion device, an area detector, a translation device (stage or platform), a controlled light source, and a computer [45]. However, for outdoor applications, several additional components are introduced to account for environmental variability and operational challenges. For instance, some of the modern outdoor HSI systems may incorporate gimbals for image stabilization, continuous calibration data acquisition setups, and different combinations of sensors that provide critical information for preprocessing and data fusion. Similarly to the systems applied indoors [46], the spectral range of the outdoor systems is defined by the spectral spectrograph and illumination type. Mobile platforms are typically equipped with a set of sensors, which may include an inertial measurement unit (IMU), RGB and depth cameras, encoders, Light Detection and Ranging sensors (LiDARs), infrared (IR) sensors, and positioning systems. The output of these sensors can be fused with HSI in both preprocessing as well as processing phases to provide fundamentally new outcomes. The schematic diagram of HSI camera integration into mobile platform is described in Figure 7.

The quality of the data by the platform is defined by the parameters of the illumination (environmental illumination or artificial illumination mounted the platform), the position of the platform and, in some applications, the stabilizer. The next step, after the raw data is recorded, is the preprocessing, which depends on the type of the sensor applied, the specific system parameters, and other sensors utilized (more details in Section 6). Material classification within a scene can be performed locally (online) using embedded processing devices on a mobile platform. enabling real-time decision making in field applications where immediate results are required. This is particularly important for navigation, where rapid material classification can enhance obstacle detection, terrain assessment, and adaptive path planning. However, online analysis requires careful power management due to the high computational demands of large classification models on edge devices, as discussed in [47] and Section 6. Alternatively, collected hyperspectral data can be analyzed offline using high-performance computers or cloud-based platforms.

### 3.1. Dispersion Device and Detector

The core of a HSI system consists of wavelength dispersion devices, which separate broadband light into multiple wavelength components using optical and electro-optical instruments [45]. The dispersion device may include prisms, gratings, filter wheels, an interferometer, acousto-optical tunable filters (AOTFs), or liquid crystal tunable filters (LCTFs) [48]. More recent models of the HSI cameras are based on Fabry–Pérot filters combined into mosaic pattern, where each pixel has a dedicated band-pass filter. Spectral filters are typically arranged in a grid, forming a repeating mosaic pattern across the detector array. The sensors utilizing this technology were applied in various research works discussing the application of HSI for mobile platforms [49,50,51] and inspection applications [52,53].

Another type of HSI sensors recently emerged are lightfield cameras, which acquire the scene using the microarray of micro lenses (lenslets) placed in front of the high-resolution sensor [54,55]. This configuration enables the simultaneous capture of multiple perspectives of a single scene. These collected post-processing flexibility, including the ability to adjust focus points after acquisition, create three-dimensional imagery, and modify viewing angles in both still images and video content after the initial capture has occurred [56].

**Figure 8:**
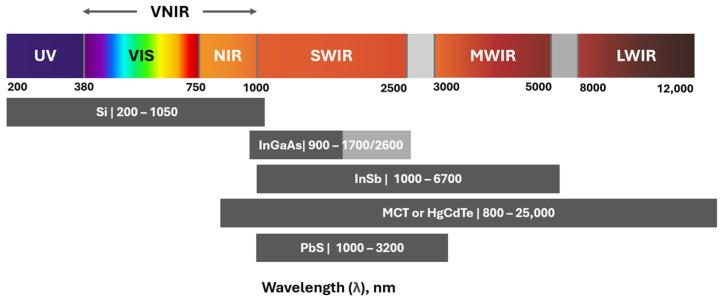
Spectral regions and associated sensor technologies across the electromagnetic spectrum.

Each detector element corresponding to a pixel in a two-dimensional (2D) detector array records the light emitted or reflected from the object and passed through the dispersion device. A hyperspectral image is generated by scanning the scene or capturing it using a snapshot imaging technique. These data are captured across several hundred spectral channels, producing a spectral response curve that provides detailed information for material characterization [17]. The material used in the detectors depends on the spectral range (See Figure 8), which is defined by the application. The materials used for detectors are silicon (Si), indium gallium arsenide (InGaAs), indium antimonide (InSb), mercury cadmium telluride (HgCdTe), and silicon doped with arsenic (Si:As) [8]. Silicon-based CCD (charge-coupled device) and CMOS (complementary metal-oxide semiconductor) sensors are the most commonly used detectors for the visible to near-infrared (VNIR) range [11]. They are widely adopted due to their high availability, lower cost, and ability to operate at room temperature.

Beyond the visible range, sensors using alternative materials cover the SWIR, MWIR, and LWIR regions. These systems typically require cooling, which increases the physical size of the sensor, as well as its cost and power consumption [57,58]. However, recently, uncooled sensors using InGaAs detectors combined with mosaic filters have emerged, enabling spectral imaging in the SWIR range [53,59].

### 3.2. Translation Device

Since hyperspectral sensors can only capture data from a limited spatial area at once, a translation device is necessary to systematically move either the sensor or the sample, ensuring complete coverage of the target area. Depending on the application, the translation device can vary. For the indoor application, static setups such as motorized stage stages, conveyors, or robotic manipulators can be used [60,61,62]. In contrast, field measurements often require moving platforms; the sensor can be mounted on mobile platforms, including planes, unmanned aerial vehicles (UAVs), mobile robots, or handheld devices.

### 3.3. Illumination

Solar radiation serves as a natural broadband illumination source for outdoor hyperspectral imaging (HSI) applications, which is why many such studies rely solely on natural sunlight. However, the intensity and spectral composition of solar radiation are strongly influenced by the atmospheric conditions, time of day, season, and geographic location. In situations where natural light is insufficient or unavailable, artificial illumination is employed. The selection of appropriate artificial light sources must consider several factors that may affect image quality, spectral accuracy, and the overall acquisition process.

The spectral output of a typical fluorescent lamp exhibits significant variations across wavelengths, characterized by distinct peaks and valleys in its emission spectrum. Two particularly prominent peaks occur at wavelengths of 540 nm and 620 nm, where energy emission reaches its high levels. This uneven spectral distribution poses a significant challenge for HSI applications, as it becomes difficult to distinguish between the inherent variations in the light source’s spectrum and the actual absorption patterns of the materials being observed [63]. In the case of high-speed imaging, fluorescent lamps may produce artifacts due to flickering.

Another light source that is applied for HSI is LED light, which provides illumination typically in UV and VNIR ranges. The main advantages of it are low energy consumption, extended operational lifetime, and low working temperatures [8]. Additionally, it is susceptible to both voltage variations and changes in junction temperature [8]. LEDs excel at directional lighting because they can emit photons in a focused direction without energy loss. LED lights can be configured in various arrangements to meet requirements defined by different systems, including spot, linear, and circular [8]. However, LEDs present several limitations for HSI applications. The light output is generally less intense compared to halogen sources, and the emission is susceptible to both voltage variations and junction temperature fluctuations.

Halogen lights provide broadband illumination when applied in the VIS and NIR part of the electromagnetic spectrum [8]. A standard configuration employs a tungsten wire filament encapsulated in a quartz glass chamber filled with halogen elements, predominantly iodine or bromine. The advantage of halogen lamps is the smoothness across the spectrum without noticeable peaks. Halogen lamps are constrained by several fundamental limitations: an abbreviated lifetime, substantial heat production, and thermal-induced spectral instability [64]. The performance of these illumination sources is also degraded by voltage-dependent output variations and mechanical vibration sensitivity [8]. The Quartz Tungsten Halogen (QTH) light bulbs are often used for outdoor HSI data acquisition [65,66,67].

An incandescent lamp operates by passing current through a tungsten filament; it provides smooth illumination in the range from UV to MWIR [68,69]. The main drawbacks of incandescent lamps are heating generation and energy inefficiency, as most of the energy generation in the IR range [69]. This type of illumination is not commonly used in spectral imaging; however, some researchers have discussed its applicability [63,70].

Another illumination tool used in imaging spectroscopy is xenon lights. A xenon lamp produces intense light that spans a wide range of wavelengths, providing consistent and even illumination in the range from UV to NIR [71]. This type of illumination delivers consistent illumination, which ensures comparability, which is crucial in terms of experimental work [72].

Lasers are directional monochromatic light sources commonly used for fluorescence and Raman measurements. A laser consists of three basic components: a resonant optical cavity/optical resonator, a laser gain medium/active laser medium, and a pump source to excite particles in the gain medium, generating light through stimulated emission [8]. Certain chemicals within samples can absorb energy from a high-energy monochromatic laser beam, which can then excite the electrons in that food to a higher-energy state. When these electrons settle back into their normal condition, they emit lower-energy light at a wide range of wavelengths, which causes Raman scattering or fluorescence emission.

### 3.4. Calibration

To mitigate the effects of ambient light on hyperspectral measurements, radiometric calibration must be applied [49,50]. This process compensates for sensor response non-uniformity and illumination variability using reference targets with known reflectance, such as Spectralon (Labsphere, Inc., North Sutton, NH, USA) panels. Spectralon is widely used due to its stable reflectance across a broad spectral range and its behavior as a Lambertian reflector, meaning it reflects light uniformly in all directions regardless of the angle of incidence [73]. In practice, a white reference pad is placed within the field of view to acquire a white reference image, enabling the normalization of reflectance values across the scene. Additionally, dark frame subtraction is commonly used to eliminate the influence of sensor dark current noise. The dark image is typically acquired using the lens cap method, where the lens is covered to block all incoming light, allowing the intrinsic noise profile to be captured and later subtracted from the measured data. Once both the white and dark reference are acquired, the reflectance can be calculated as discussed in [74].

### 3.5. Stabilizer

In terms of outdoor HSI, in some cases, the stabilization and control of orientation are often required during data acquisition. Gimbal systems effectively mitigate artifacts caused by both vibrations and motion. These devices precisely control the pointing direction and FOV of the sensor. High-precision control is essential, as both vibrations and platform motion can significantly degrade data quality. However, it is crucial to emphasize that the use of gimbals has an impact on power consumption.

## 4. Acquisition Modes

The fundamental way to classify the HSI systems is by using image acquisition modes, which describe how the spectral and spatial information is recorded. They influence system applicability across various contexts, ranging from precision laboratory measurements under controlled conditions to dynamic data collection in field environments. To collect the 3D hypercube, there are four hyperspectral data acquisition modes used: point scanning (whisk broom), line scan (push broom), area scanning (wavelength or spectral scanning), and snapshot (non-scanning) [46].

### 4.1. Point Scanning

**Figure 9:**
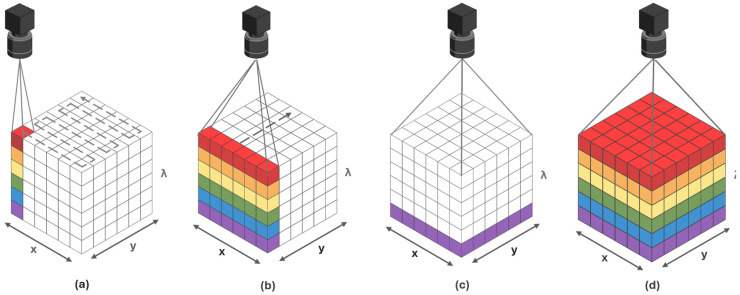
HSI acquisition modes: (**a**) point scanning, (**b**) line scanning, (**c**) wavelength scanning, and (**d**) snapshot.

Point scanning (see Figure 9a), sometimes referred as imager whisk broom scanning, is where each point is acquired individually. Spatial resolution can be achieved in different ways and the ways to achieve that are selected depending on the application. In the lab setups, both the sample and detector can be moved in a zigzag pattern on the spatial dimension (x and y) [75]. In remote sensing applications, a rotating mirror is used to scan different points on the axis perpendicular to the direction of platform travel. This is why such sensors are sometimes called across track scanners [76]. The main advantage of this mode is the ability to obtain a high spectral resolution. However, as gathering point-by-point spectra takes an extensive amount of time, spatial resolution may be low. Despite this limitation, whisk broom scanning systems are adaptable regarding spectrum ranges, raster width, sample size, and optical technique implementation [38,77]. Point scanning is widely used in microscopic imaging, where the acquisition time is not the limiting factor [78].

### 4.2. Line Scanning

In the case of line scanning (see Figure 9b), a push broom system captures spectral information across all wavelengths for each point within a linear array of pixels in a single acquisition. With this technology, the material moves in the y-direction while spatial and spectral information accumulates along the x-direction. Due to its straightforward design requiring only linear sample movement and no filter adjustments, this method integrates effectively with high-speed systems [78].

### 4.3. Area Scanning

Area scanning systems (see Figure 9c), also known as wavelength or spectral scanning devices, utilize a two-dimensional detector array to capture the complete spatial scene through multiple exposures, each at a different wavelength. The system operates without moving either the sample or the hyperspectral camera, and it relies on either a filter wheel or a tunable filter to select specific wavelengths during data acquisition. This area array sensor approach is particularly well-suited for MSI applications, where imaging occurs at a limited number of selected wavelengths and is not suitable for acquiring moving environments [79].

### 4.4. Snapshot Camera

Single shot mode, also called snapshot (see Figure 9d), collects both spectral and spatial information about the scene in a single measurement period [80]. Compared to previously mentioned modes, snapshot is a non-scanning technique. Modern snapshot sensors can deliver high frame rates, enabling real-time or near-real-time HSI [81,82]. Technology has significantly evolved over the last decades, mainly supported by the developments in sensor design. One of the advantages of these sensors is the absence of moving parts, which increases the robustness of the system. However, due to their lower spectral and spatial resolution, these sensors require substantial computational resources alongside sophisticated processing algorithms to maximize image quality [46].

### 4.5. Applicability of Modes for Mobile Platforms

Parameters dedicated to the acquisition, specified in Table 1, significantly influence the application scenario of mobile platform-based HSI systems. Snapshot cameras can be applied for navigation purposes due to their low sensitivity to motion, large FOV, and large spatial resolution. For inspection tasks, three alternative scanning methods offer specific advantages. In general, the main disadvantage of scanning techniques is the motion-related artifacts [83]. Thus, these scanning modes can be applied when the motion is illuminated. Point scanning enables high spectral resolution sampling of stationary configurations; this type of scanning can be used for calibration purposes as well [50,66]. Line scanning provides measurements suited for the systematic inspection of areas while being dependent on platform movement. Area scanning delivers comprehensive data collection for static scenes with low spectral resolution.

## 5. Applications of HSI on Ground Mobile Platforms

In recent years, the prospects for using hyperspectral cameras have begun to be actively studied in various ground mobile platforms. Those studies cover the integration of spectral imaging sensors into autonomous vehicles, unmanned ground vehicles (UGVs), and tracked and legged robots. In terms of application, existing solutions can be divided into two main categories: navigation and inspection.

In the case of navigation, the information the sensor receives is used to control the movement of the platform, including path and speed planning, etc. This category also includes the use of Advanced Driving Assistance Systems (ADAS) applied in modern vehicles. Those systems require near-real-time or real-time feedback processing of HSI sensor data, which is crucial for decision making. Both spectral and spatial resolution play important roles in this case. In the case of inspection tasks, HSI is used to obtain information regarding the environment. Unlike navigation applications, inspection tasks can utilize post-processing of collected data, reducing real-time requirements.

The following sections aim to discuss the application of HSI in ADAS, navigation, and inspection. Although ADAS systems belong to the navigation sub-category, it was decided to write about that separately due to their specific use in conventional vehicles, which defines their unique operational conditions.

### 5.1. Autonomous Navigation

The application of HSI for navigation requires high acquisition and processing speed, contributing to the latency. Typically, RGB cameras used for autonomous driving operate at 30 frames per second [84]. The exploration of HSI for navigation purposes became mainly possible thanks to the availability of lightweight and relatively affordable snapshot hyperspectral cameras capable of delivering high framerates. These sensors utilize silicon-based detectors operating in the VNIR range, making them both cost-effective and practical for navigation purposes.

#### 5.1.1. ADAS

Modern autonomous vehicles rely on a comprehensive sensor suite, including RGB cameras, ultrasonic sensors, radar, and LiDAR, with infrared cameras enabling nighttime operation [85]. Despite these advanced sensing capabilities, several critical challenges remain in environmental perception and interpretation. A Congressional Research Service Report [86] emphasizes the dependency of autonomous vehicles on road infrastructure, particularly markings and traffic signs, highlighting the need for more resilient sensing systems that maintain reliability when traditional visual cues degrade. Rural and off-road environments present additional complexities, where terrain features like mud, potholes, and loose gravel can impede vehicle operation. This has spurred research into spectral imaging for enhanced terrain understanding [87]. The next key challenges might be highlighted:

- Dependence on road infrastructure, including markings and traffic signs;
- Rural environments and offroad navigation;
- Discrimination between visually similar objects;
- Adverse weather conditions.

To solve these challenges, the researchers investigate the perspective of integrating other sensors. In the case of spectral imaging, it is possible to say that the first attempts to go beyond the visible with ADAS systems started with the application of IR sensors, which were primarily used for nighttime scenarios. In [88], the authors compared the traditional RGB camera with NIR, SWIR, and LWIR IR sensors for driving-related scenarios like pedestrian detection, road marking, and traffic sign observation in fog.

In 2017, researchers from Active Vision Group (AGAS) of the University of Koblenz-Landau introduced both dataset and methodology for drivability estimation using HSI cubes acquired from a moving vehicle. Their experimental setup consisted of two cameras (XIMEA GmbH, Münster, Germany), chosen to cover both VIS (16 bands) and NIR (25 bands) parts of the electromagnetic spectrum, enabling comprehensive terrain analysis [83]. The publicly available HyKo1 dataset was focused mainly on terrain drivability estimation with manual annotations across four classes: drivable, rough, obstacle, and sky [49]. The per-pixel classification was performed using random forest and Gaussian Naïve Bayes classifiers, applied to both raw reflectance data and data compressed using principal component analysis (PCA) [83]. For the VIS hyperspectral data, random forest achieved precision and recall values above 75% across all terrain classes for both raw and PCA compressed inputs. In contrast, Gaussian Naive Bayes (GNB) showed significant variability, with the obstacle class achieving only 38% recall, while the sky class reached 90% recall on raw data. For the NIR data, the camera was directed downward, capturing only the terrain ahead of the vehicle, thus excluding the sky class. In this configuration, GNB failed to detect the obstacle class, whereas random forest performed more robustly, achieving 70% precision and 56% recall for obstacles. The results demonstrated the effectiveness of spectral imaging for drivability classification. It also indicated that parts of the scene, including chlorophyll, were well separated due to absorption at 675 nm wavelength. While the results demonstrated the effectiveness of HSI for drivability classification, pixel-wise classification occasionally produced noise due to limited neighborhood context.

This challenge was addressed a year later in one of the following papers using HyKo2. The subsequent version of the dataset expanded its annotation scheme to include three annotation types: material classes, semantic classes, and drivability classes. The methodology was enhanced by implementing context-aware fully connected conditional random fields [89]. This improvement yielded better classification results with fewer outlier pixels and enhanced separation of vegetation regions.

Next, in 2019, the potential of unsupervised and supervised deep learning methods for pixel-level classification was investigated in [90]. The two-step pipeline consisted of compression and classification. Initially, the normalized reflectance data were compressed into the latent space using a regularized deep autoencoder. After that, the pixel classification was performed using different architectures like BiseNet, MobileUNet, FRRN-A, Enocder-Decoder, and FRRN-B. The study showed that classifying NIR data compressed by the autoencoder using BiSeNet achieved the best performance, yielding an IoU value greater than 0.5.

Publications from the AGAS group [49,89,90,91] confirmed that HSI can be used for semantic scene understanding in autonomous driving scenarios. The research provided two comprehensive datasets combining data from dual HSI cameras, a Velodyne LiDAR, a spectrometer, and an RGB camera (HyKo1) acquired in Koblenz. Although the data was collected, the publication did not consider the fusion of obtained data. The datasets cover scenes illustrating applications in inner-city traffic environments with well-established infrastructure and countryside roads, captured on 16 June (HyKo1) and 16 November (HyKo2) 2016. However, their work did not address real-time processing capabilities.

The exploration of HSI applications in ADAS continued in 2021 with the introduction of the HSI-Drive v1.1 dataset by researchers at the University of the Basque Country [92]. This dataset featured images captured during spring and summer under varying weather conditions (sunny, cloudy, foggy, and rainy/wet) and lighting conditions (dawn, full daylight, and sunset). The HSI camera (Photonfocus AG, Lachen, Switzerland) used in their tests covered the range of 600–975 nm, producing 25 bands. Annotation was performed based on material classes of objects within the scene. In this dataset, similarly to HyKo datasets, some pixels within the scene were not assigned classes during the annotation process, particularly at material boundaries where definitive categorization was challenging or ambiguous due to spectral mixing. This dataset added new classes, such as painted metal and glass/transparent plastic. The data were acquired at 11 frames per second to manage memory consumption. Prior to classification, the separability of the classes was evaluated using the Jeffreys–Matusita distance. High separability values were obtained for “Sky” and “Road” classes, whereas pairs of “Painted metal” and “Unpainted metal”, as well as “Painted metal” and “Pedestrian/Cyclist”, demonstrated low separability. Artificial neural networks (ANN) with two hidden layers were employed for analysis. Four experiments were conducted to examine various class combinations for classification. The researchers noted that classes with a small number of representatives achieved low classification scores. In some cases, false detections of pedestrians were observed on highways.

Subsequent research shifted focus toward evaluating existing segmentation models and deploying them on embedded platforms [47]. Two classification methods, the ANN and the U-Net-based fully convolutional network (FCN), were tested. The ANN, with three hidden layers, was trained using all 25 spectral bands for pixel-wise classification, as well as pseudo-RGB images constructed through orthogonal space projection [93]. Using complete spectral information produced better classification outcomes. Next, the modified U-Net with different hyperparameters was tested. For the processing of the complete hypercube, it demonstrated strong performance in three-class experiments (“Road”, “Road marking”, and “Non-drivable”). However, the model struggled with classification accuracy in challenging conditions such as shadowed areas and scenes with overlapping thin metallic structures. In five-class tests (“Road”, “Road marking”, “Vegetation”, “Sky”, and “Non-drivable”), the algorithm was able to detect smaller objects like traffic signs on the non-drivable parts of the scene and identified the regions of vegetation. Similar tests with pseudo-RGB showed significantly reduced performance. Furthermore, the model was deployed on embedded devices, and the corresponding evaluation is discussed in Section 6.

Released in 2022, the HSI-Drive 2.0 four-season HSI video dataset opened new avenues for research exploration [51]. An increased training data size led to enhanced segmentation results. Among different weather conditions, the solution demonstrated optimal performance under cloudy conditions, benefiting from the diffused, homogeneous illumination. In contrast, the lowest performance was recorded under sunny conditions, where scenes displayed significant illumination contrasts between heavily shadowed areas with low reflectance and overexposed sunlit regions, posing substantial challenges for accurate processing. Rainy weather did not pose significant challenges for the classification, struggling only with road marking. The algorithm performed well on a video featuring a complex road scene during a sunny winter morning, characterized by frontal sunlight and severe glares on the tarmac. The data demonstrated varying saturation levels across 25 spectral bands, with band 24 having the lowest number of saturated pixels, potentially showing the benefits of rich spectral resolution compared to RGB. More details regarding the influence of the environment on measurement are discussed in Section 6.

Autonomous driving relevant urban environment classification was also addressed in two datasets acquired in Shanghai: Hyperspectral City v1.0 and v2.0 [94,95]. These datasets were featured in the Physics-Based Vision meets Deep Learning challenges at ICCV workshops in 2019 and 2021. The datasets stand out for their exceptional data quality, combining remarkable spectral resolution across 125 bands with a high spatial resolution of 1400 × 1800 pixels and contributing to further investigations of the topic [96].

Finally, in 2024, the Hyperspectral Semantic Segmentation benchmark (HS3-Bench) was introduced by researchers at the University of Koblenz-Landau [97]. Their paper introduced the description of procedures required for achieving comparable evaluation of HSI segmentation, followed by their implementation using HyKo2, HSI-Drive v2. 0, and Hyperspectral city v2.0. One of the important conclusions addressed in the paper was the need for a detailed analysis of the impact of adding additional HSI channels and increasing RGB training data on segmentation performance [74].

#### 5.1.2. Offroad Navigation

The application of HSI is not only investigated in well-structured environments suitable for autonomous vehicles. In this case, the term “drivability” commonly used in HSI applications dedicated to ADAS, is replaced by “terrain traversability”. In recent years, the traversability estimation using volumetric representations of complex environments was actively studied [98]. However, in [99] Jakubczyk et al. highlighted that in some cases, having information regarding the terrain geometry is not sufficient, as traversability may significantly depend on the material of the ground and its structure. For example, from an object geometry point of view, rock, tall grass, and small bushes are obstacles having the exact physical dimensions. The navigation system integrated into the ATENA vehicle (Łukasiewicz Research Network–Industrial Research Institute for Automation and Measurements PIAP, Warsaw, Poland) included three VLP-16 LiDARs (Velodyne Lidar Inc., San Jose, CA, USA), five acA1920-48gc (Basler AG, Ahrensburg, Germany) cameras, a MTi-G-710 (Xsens Technologies B.V., Enschede, The Netherlands) IMU, and a Q285 (Cubert GmbH, Ulm, Germany) HSI camera (450–950 nm). The information from LiDARs was used to build a hexagonal grid-based map enhanced with semantic labels corresponding to specific materials. The study considered five classes: ground road, forest road, asphalt road, grass, and forest. Labeling the scene was diving into the grid and assigning the label to each cell. The classification in this study employed a non-parametric supervised nearest-neighbor approach, where the spectral distribution of cells with unknown labels was compared against distributions with known labels from the dataset using the chi-square distance metric to assign terrain labels. Compared to ANN-based solutions, the proposed method is non-parametric and facilitates the interpretability of results. Similarly to [89], the neighborhood semantic information is considered for contextual adjustments. After that, semantic information is fused with a 2.5D map generated by LiDAR to generate a map of environments, serving as the cost function. Path planning utilizes the cost of driving over different terrains, which depends on the type of material present within each map cell.

**Figure 10:**
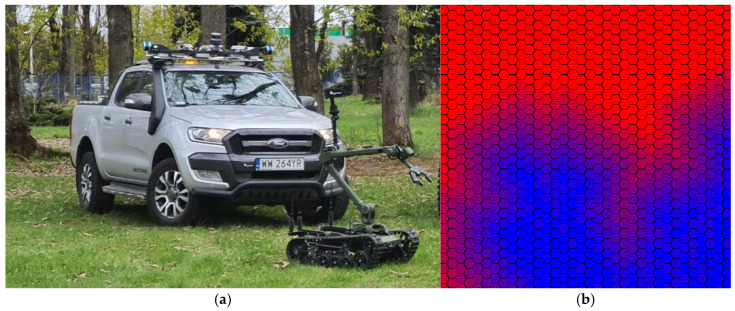
(**a**) Łukasiewicz–PIAP PATROL UGV platform (Left) and Łukasiewicz–PIAP ATENA platform (right) [100]; (**b**) map of maximum permissible speed (80 km/h, red color; 10 km/h, blue color) [100].

Further development of the idea with the generation of a hexagonal traversability map resulted in a new platform-centric approach to environment interpretation [100]. The calculation of the map, in this case, considers both the material of grid cells and the parameters of the specific platform [100]. The study included the generation of custom traversability maps for PIAP PATROL UGV (Łukasiewicz Research Network—Industrial Research Institute for Automation and Measurements PIAP, Warsaw, Poland) (100 kg) and PIAP ATENA (2200 kg) (Łukasiewicz Research Network–Industrial Research Institute for Automation and Measurements PIAP, Warsaw, Poland) platforms (Figure 10a). The methodology assumed that material properties correlate with different road frictions and defined the speed of the vehicle to be selected. For that reason, each cell in the grid was assigned maximum velocity limits (Figure 10b). Moreover, potentially dangerous transition regions with materials having different friction coefficients were considered, taking into account platform parameters. Researchers have highlighted that the environment’s multilayered nature can significantly affect terrain traversability. Although the surface may present as grass, wheels or tracks actually contact underlying layers of mud or clay. These critical subsurface layers, which truly determine traversability, might become apparent only during the actual traversing process.

**Figure 11:**
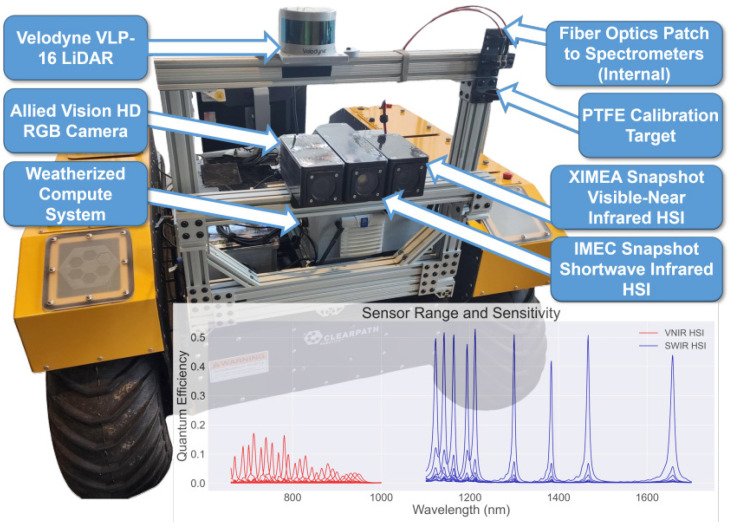
Hyper-Drive3D system platform [59].

The application of HSI was tested on the Warthog (Clearpath Robotics, Kitchener, ON, Canada) off-road mobile platform (see Figure 11) [50]. It resulted in the creation of the vehicle-centric HyperDrive dataset, which includes 12,874 scenes, 500 of which are finely annotated. Compared to previous studies, the sensor setup on this platform incorporated an uncooled InGaAs sensor (IMEC, Leuven, Belgium) operating in the SWIR range of 1100–1700 nm. Combined with a VNIR (IMEC, Leuven, Belgium) camera covering the visible to near-infrared spectrum (660–900 nm), the HSI acquisition system provided 33 spectral channels across a wide range. The data collection was conducted at various times of the day, such as sunrise, midday, late afternoon, and sunset, capturing natural variations in sunlight intensity and image brightness. The testing encompassed a range of terrains, including paved roads, hiking trails, and dirt paths. The developed system was later used for the wildfire risk assessment using the forest biomass evaluation [59].

In their previous work, the researchers used a spectrometer covering the 350–1150 nm range, along with additional sensors, mounted on a Jackal UGV (Clearpath Robotics, Kitchener, ON, Canada) to classify terrain materials near the tire contact points [66]. The authors developed the Visual and Spectral Terrain Classification (VAST) sensor array for enhanced terrain classification. The system consists of a set of sensors mounted on a tray positioned in front of each wheel, facing downward to capture local road patches. The VAST sensor array includes a QTH light bulb by Thorlabs (Thorlabs Inc., Newton, NJ, USA) for consistent broadband illumination, a BLUE-Wave (StellarNet, Inc., Tampa, FL, USA) miniature VNIR spectrometer, and an 8-megapixel USB camera (Arducam Technology Co., Ltd., Shenzhen, China) equipped with a macro lens. Using a fusion of spectral, visual, and IMU data in a neural network architecture, the system achieved a terrain classification accuracy of 99.98%.

**Figure 12:**
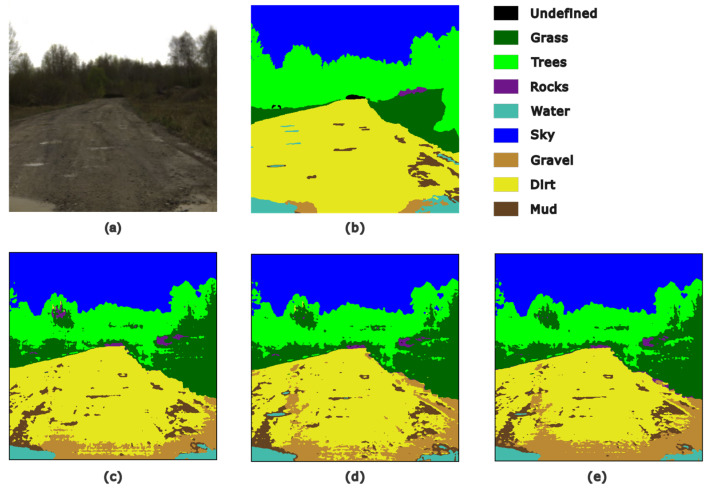
Results of unstructured terrain scene classification using spectral–spatial CNN and min–max pooling: (**a**) RGB color composite image of HSI cube; (**b**) ground truth; (**c**) classification result for 9 bands; (**d**) classification result for 16 bands; (**e**) classification result for 25 bands [103].

The unstructured terrain analysis was also addressed by researchers from Tallinn University of Technology [101,102,103]. In [101], Liyanage et al., investigated the application of HSI images to facilitate the semantic segmentation of RGB images using the Resnet18 network. The images were acquired using the Specim IQ V10E (Specim, Spectral Imaging Ltd., Oulu, Finland) handheld HSI camera acquiring scenes in VNIR (400–1000 nm) with a spatial resolution of 512 px × 512 px. The number of the bands was reduced using the min–max pooling method previously developed by researchers in [102]. For training, pixel-wise annotated false RGBs were used. The study analyzed the performance of the model across different classes across scenes with different terrain structures. It is reported that, in some cases, the model misclassified grass and wet fallen leaves on the stream. The accuracy across different scenes described varied from 77.3% to 81.9%. Figure 12 illustrates classification using spectral–spatial CNN and min–max pooling discussed in [103].

#### 5.1.3. Autonomous Driving Relevant Scenarios

While many publicly available research works and datasets were not specifically designed for autonomous driving applications, their methodologies and findings can be valuable for evaluating HSI in various scenarios. These resources often address fundamental challenges such as material classification, illumination variation, and atmospheric effects, which are equally relevant to autonomous navigation systems.

For instance, in 2023, Korean researchers in [104] conducted a study on black ice detection using HSI technology. The research was performed in an urban environment using a HS-CL-30-V10E (Specim, Spectral Imaging Ltd., Oulu, Finland) sensor, with data collection occurring during morning hours to optimize detection conditions. The study examined two distinct black ice samples: naturally formed ice on asphalt pavement and a controlled sample prepared by researchers and contained within a black box. Images were captured at three different distances (10, 20, and 30 m) to evaluate detection capabilities across varying ranges. The imaging system operated at a spatial resolution of 1332 × 854 pixels, and principal component analysis (PCA) was employed to reduce the spectral dimensionality to 10 principal components, enhancing computational efficiency while maintaining essential spectral information. The results demonstrated successful black ice classification at distances of 10 and 20 m, with high detection accuracy. However, detection performance notably degraded at 30 m, suggesting a practical distance limitation for this technological approach [104].

HSI can also be used to detect car headlights. Three types of headlamps were discovered: LEDs of E-class (Mercedes-Benz Group AG, Stuttgart, Germany), high-intensity discharge of Grandeur-HG (Hyundai Motor Company, Seoul, Republic of Korea), and halogen lamps of Sorento (Kia Corporation, Seoul, Republic of Korea). In the study, the researchers used a ImSpector V10E (Specim, Spectral Imaging Ltd., Oulu, Finland) sensor (400–1000 nm); the spatial resolution of the images finally used was 200 × 350 pixels. Researchers successfully distinguished both the front and rear lights of vehicles in the urban scene [105].

#### 5.1.4. HSI Datasets for Mobile Navigation and ADAS

The various studies that were conducted produced public HSI datasets containing scenes relevant to mobile platforms, recapped in Table 2. However, while working with those datasets, several aspects should be considered. In [51], it was noted that the intraclass variability, in some cases, may influence the performance of the model. For example, classes like “Road mark” usually correspond to specific materials, whereas the class “Pedestrian” has significant variability, as it includes subclasses like motorcyclists, cyclists, animals, and passers-by. Similar findings regarding the pedestrian classification were discussed in [106]. Another example of intraclass variability is correlated with seasonal variation. In [99], Jakubczyk et al. demonstrated that the grass had different spectral signatures in spring and autumn. Quite often, the interclass variability comes from complex scene labeling being labor-intensive and complicated by the complex visual differences between materials and their high degree of mixing. For example, this was the reason why, in [102], Liyanage et al., in addition to “dry matter”, “mud”, and “sand”, annotate the subclass “gravel”.

Secondly, researchers use different approaches to annotate their data. In the case of acceptable semantic labels, the pixels of the scene are associated with the dedicated classes using masks. Coarse labels, in contrast, may have next labeling approaches:

- Fine semantic labels: classis approach [50];
- Coarse semantic labels: leaving the edges unlabeled [49,51,92];
- Grid labels: splitting the image into a grid of patches [99].

The main benefit of coarse labeling is saving time, as annotating using acceptable labels is time intensive. However, the quality of the labels quite often defines the segmentation results. This issue was addressed in [96], where a weakly-supervised HSI semantic segmentation system uses spectral information to refine coarse labels. Additionally, the researchers applied the t-SNE method to analyze the class separability of classes in HSI and RGB images. In the case of HSI, the precise boundary between the classes and continuous distributions could be observed, highlighting its applicability.

The types of classes used for labeling can also be used to characterize the dataset. Generally, there are three types of classes: semantic classes, material classes, and drivability classes. In the case of labeling from the point of view of the drivability of a particular environment, the focus shifts towards the parameters of the platform and might not be unified. The drivability of the environment also depends on the parameters of the platform considered in the specific study associated with the dataset. For semantic classes, the priority shifts to analyzing spectral signatures relevant to certain objects, such as vehicles, which combine signatures of painted metal, glass, and plastic. However, objects made of similar materials might get confused during classification. Material-based classification focuses on identifying the specific materials composing objects. In this case, both cars and traffic signs might be referred to the same class. In some datasets, the scenes are labeled using all three levels [49]. This multi-layer annotation approach enables a more sophisticated analysis of the scenes. The difference in labeling approaches reflects distinct end goals in the scene classification purposes. Generally, researchers utilize three approaches for scene annotation in their datasets:

- Drivability-based annotation: Labels are based on either drivable or non-drivable;
- Object-based annotation: Labels are based on correspondence to a predefined object category (e.g., car, building, road);
- Material-based annotation: Labels are based on the type of surface material (e.g., concrete, painted metal, asphalt, paint).

The lack of standardization in datasets was addressed by Hanson et al. in [50]. To overcome this, the ATLAS label set [107] was suggested to facilitate the consistency of the dataset structure. In this case, each hypercube is described using the biome, season, time of the day, season, and weather. Instance labels are used to characterize the features of the scene, and the highest-level features include those that can be further decomposed into lower-level features. For example, “obstacle” class can be split into subclasses like “vehicle”, “infrastructure”, and “road signage” [50].

A fundamental difference among the available datasets lies in their geographical acquisition context. The datasets have been collected in a variety of regions, including Europe (Germany [49], Spain [51,92], Estonia [108]), Asia (China [94,95]), and North America (the United States [50]). This diversity introduces significant variability in the spectral characteristics of the materials present in each scene. Natural materials such as vegetation, soil, and minerals are particularly sensitive to regional factors including biome type, seasonal cycles, and environmental conditions such as humidity, temperature, and illumination. In contrast, man-made objects such as vehicles, road surfaces, and urban infrastructure tend to exhibit more consistent spectral responses across regions, as they are commonly constructed using standardized industrial materials like asphalt, painted metal, and plastic.

For data acquisition, the researchers have used different hardware setups, which included either only a HSI or the HSI camera combined with other sensors. In studies such as [51,92,94,95,108], the datasets include both hypercubes and annotations; in some cases, an RGB image of the scene is also provided. In contrast, the Hyper-Drive dataset includes not only raw HSI scenes, RGB images, and corresponding labels, but also measurements of solar illumination for each hypercube, captured using two spectrometers. What makes the HyKo1 dataset stand out from others is the fact that the HSI data are accompanied by scene acquired with acA2040-25gc RGB camera (Basler AG, Ahrensburg, Germany), HDL-32E (Velodyne Lidar Inc., San Jose, CA, USA) LiDAR point cloud, Qmini (Broadcom Inc., San Jose, CA, USA) spectrometer and GPS measurements. However, in the subsequent version, HyKo 2, RGB scene images and GPS information are omitted.

Some datasets provide only coarse labels [95,96], while others use fine, pixel-wise annotations that emphasize detail [108]. Coarse labeling is often chosen either to avoid highly mixed pixels by ignoring border regions or due to time constraints involved in detailed annotation. Fine annotations, on the other hand, can significantly improve prediction accuracy by providing more precise ground truth data.

Another important factor to consider is the type of HSI sensor used for the measurements. The three main parameters that influence the quality and applicability of the data are spectral resolution, spectral range, and spatial resolution.

Different devices with varying spectral resolutions have been used across the datasets. The dataset with the highest number of bands is [108], which contains 204 bands; [94] and [95] each contain 129 bands. These datasets offer a high capacity for material discrimination due to their high spectral resolution. Most of the other datasets were acquired using snapshot cameras based on Fabry–Pérot mosaic filter arrays with up to up to 25 spectral bands. Because of the design of the mosaic filter, the spatial resolution of the raw data acquired at a specific wavelength is inversely proportional to the size of the individual filter elements. This means that to achieve higher spatial resolution, preprocessing techniques such as demosaicing or interpolation are required.

In terms of spectral range, the majority of datasets were acquired in the VNIR part of the electromagnetic spectrum. However, the Hyper-Drive [50] dataset is notable for its coverage of the SWIR region. It was recorded using an uncooled InGaAs sensor (IMEC, Leuven, Belgium), which allows for the detection of spectral features not accessible in the VNIR range.

**Table 2:** Public HSI datasets with scenes relevant to mobile platforms.

Dataset	Year	Sensor	Manufacturer	Spectral Range	Number of Bands	Mode	Number of Classes
HyKo 1 [49]	2017	MQ022HG-IM-SM4X4-VIS	XIMEA GmbH, Münster, Germany	470–630	15	Snapshot	5 classes (drivability)
		MQ022HG-IM-SM5X5-NIR	XIMEA GmbH, Münster, Germany	600–975	25	Snapshot	
		Qmini Wide	Broadcom Inc., San Jose, CA, USA	225–1000	2500	Point scan	
HyKo 2 [49]	2017	MQ022HG-IM-SM4X4-VIS	XIMEA GmbH, Münster, Germany	470–630	15	Snapshot	11 classes (semantic) 9 classes (materials) 5 classes (drivability)
		MQ022HG-IM-SM5X5-NIR	XIMEA GmbH, Münster, Germany	600–975	25	Snapshot	
		Qmini Wide	Broadcom Inc., San Jose, CA, USA	225–1000	2500	Point scan	
Hyperspectral City v1.0 [94]	2019	Not specified	LightGene, Nanjing, China	450–950	129	Snapshot	10 classes (semantic)
Hyperspectral City v2.0 [95]	2021	Not specified	LightGene, Nanjing, China	450–950	129	Snapshot	19 classes (semantic)
HSI road [109]	2020	MQ022HG-IM-SM5x5	XIMEA GmbH, Münster, Germany	680–960	25	Snapshot	2 classes (semantic)
HSI drive v1.1 [92]	2021	MV1-D2048x1088-HS02-96-G2	Photonfocus AG, Lachen, Switzerland	600–975	25	Snapshot	10 classes
HSI drive v2 [51]	2022	MV1-D2048x1088-HS02-96-G2	Photonfocus AG, Lachen, Switzerland	600–975	25	Snapshot	10 classes
Hyper-Drive [50]	2023	Not specified	IMEC, Leuven, Belgium	660–900	24	Snapshot	5 classes (semantic) 10 classes (materials)
		Not specified	IMEC, Leuven, Belgium	1100–1700	9	Snapshot	
		Pebble VIS-NIR	Ibsen Photonics, Farum, Denmark	550–1100	256	Point scan	
		Pebble NIR	Ibsen Photonics, Farum, Denmark	950–1700	128	Point scan	
Hyperspectral image dataset of unstructured terrains for UGV perception [103,108]	2024	IQ	Specim, Spectral Imaging Ltd., Oulu, Finland	400–1000	204	Push broom	9 classes (semantic)

### 5.2. Inspection

Alongside navigation, spectral cameras are used for inspection and monitoring purposes across various fields, enabling detailed scene analysis that conventional systems cannot achieve. In agriculture, spectral imaging on mobile platforms has been extensively studied for applications including crop health monitoring, yield prediction, disease detection, and precision farming [110,111]. Moreover, several comprehensive reviews have addressed the technical aspects of implementing HSI [4,15,16]. In many fields, inspection is a process that demands expertise from the inspector. Data acquisition using mobile robots is not only a way of removing humans from the monotonous and often risk-associated tasks in challenging environments, but it also provides detailed and well-documented information that can be used for efficient asset management and research.

#### 5.2.1. Search and Rescue

Obtaining information in degraded visual environments (DVEs) is crucial in rescue operations, where conditions such as haze or smoke can affect visibility. For that reason, the application NIR spectral cameras (IMEC, Leuven, Belgium) was tested for firefighter tracking, as discussed in [52]. Integration perspectives were evaluated through real-world applications simulating such scenarios. The study proposed a two-stage detector composed of NanoDet-Plus and HybridSN models. Initially, the NanoDet-Plus was to detect the firefighters across the last three bands of the NIR in the spatial domain. These bands were selected for their ability to leverage the spatial characteristics of the scene, due to the enhanced penetrating ability of dedicated wavelengths. After that, the HybridSn model was used to perform pixel-wise classification within the identified region of interest.

Another goal relevant to rescue operations is victim detection in post-disaster situations or emergency cases [112]. To address this problem, investigators in [113] utilized the Altum MicaSense (AgEagle Aerial Systems Inc., Wichita, KS, USA) MSI camera capturing six bands. The data acquisition took place in outdoor and indoor environments resembling post-disaster scenes. The camera system was mounted on a remotely operated quadruped robot, enabling the safe and efficient exploration of hazardous areas.

#### 5.2.2. Mines

**Figure 13:**
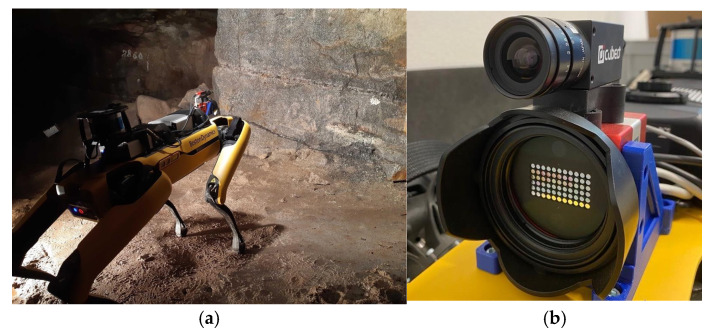
HSI system integration on quadruped platform: (**a**) Boston Dynamics Spot robot with sensing payload in mine; (**b**) Cubert X20P HSI light field camera attached to the robot with 3D printed mount [67].

The integration of spectral imaging and legged robots or their hybrids is gaining attention, as such platforms can operate in environments where traditional wheeled or tracked platforms cannot operate. In [67], The Spot (Boston Dynamics, Waltham, MA, USA) versatile robot with the X20P (Cubert GmbH, Ulm, Germany) HSI camera and VLP-16 LiDAR (Velodyne Lidar Inc., San Jose, CA, USA) in the autonomy payload was tested in underground mining environments for mineral exploration (see Figure 13). Tests were done in both the lab as well as in Zinnwald/Cinnovec visitor mine in Germany. The features of HSI data were extracted using minimum noise fraction (MNF) and PCA; next, supervised NFIDR and fully constrained least squares (FCLS) were applied providing interpretable results. Also, it was summarized that lightweight HSI cameras might be beneficial for the platforms sensitive to the maximum payload weight.

**Figure 14:**
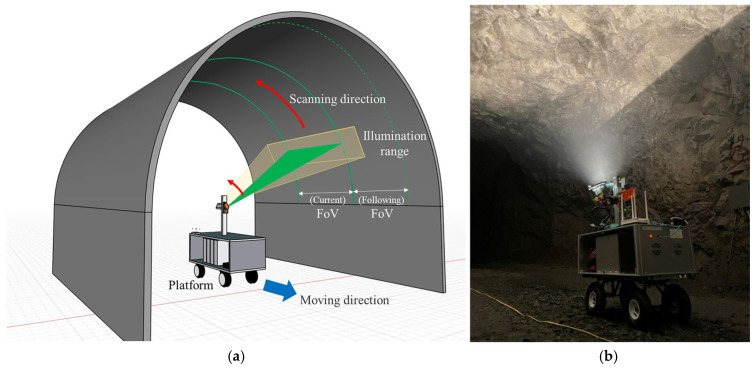
The tunnel coaxial 3D hyperspectral scanning system: (**a**) The HSI camera and the light source rotate coaxially with respect to the tunnel maintaining homogeneous light in the illumination range (marked yellow) of the FOV of the sensor (marked green) across the measurements; (**b**) system performing measurements in Gwan-In Magnetite Mine, Pocheon, Republic of Korea [65].

Similarly, in [65], the proposed coaxial 3D hyperspectral scanning setup mounted on a cart-type platform (Figure 14) facilitated the analysis of the ceiling on the mine tunnels and shafts, which are otherwise difficult for mine workers to directly inspect. The main feature of the system was the design of a coaxial push broom HSI camera synchronized with a rotational illumination source, enabling consistent measurements. To reconstruct the 3D model of the environment, a set of multiple sensors was used, combining stereo camera ZED (Stereolabs Inc., San Francisco, CA, USA), MW-AHRSv1 IMU (NTREX Corp., Incheon, Republic of Korea), and RPLIDAR-A3 LiDAR (Shanghai Slamtec Co., Ltd., Shanghai, China). Then, the hyperspectral images were projected on a generated half-cylindrical model of the tunnel plane. However, it was noted that fine vibrations caused by ongoing mining activities in real-life applications could potentially influence measurement accuracy by introducing additional requirements for IMU and post-processing. The system might also provide helpful information about vulnerable areas inside the mine.

#### 5.2.3. Infrastructure Inspection

Several research works investigated the use of spectral imaging for infrastructure inspection, particularly in determining the condition of road surfaces. One such study, conducted in Los Angeles in the period of 2001 to 2004, investigated the use of HSI-sensed data to evaluate road quality indicators like the Pavement Condition Index (PCI) [114,115]. The study combined ground spectrometry in 450–2450 nm, imaging spectrometry in the 450–900 nm range (airborne platform mounted), and in situ pavement surveys to examine spectrum changes caused by asphalt aging and deterioration. The findings achieved using statistical analysis methods revealed a distinguishable relationship between hydrocarbon-related absorption characteristics and the aging process of asphalt surfaces. However, image spectrometry effectively identified good roads but was less reliable in analyzing seriously eroded surfaces. Later, the application of spectral imaging for road condition estimation was examined by different researchers [116,117,118,119]. For example, in 2021, an Asphalt Crack Index (ACI) was suggested utilizing the 450–550 nm range [120].

Spectral imaging can also provide valuable input regarding the condition of the building and provide information about the weathering of materials [121]. By acquiring the spectral signatures of materials in different weathering phases, it is possible to build the classifier for building condition estimation. Zahiri et al., in [122], discuss applying multi- and hyperspectral cameras for façade material classification. Partial least squares discriminant analysis (PLSDA) and support vector machine (SVM) classifiers were used to classify brick, mortar, stone, painted window frames, and rendering. Potentially, such information could contribute to building information modeling [123].

The inspection of power grid components is crucial for preventing blackouts and maintaining reliable electricity supply. The study [124] explored the application of HSI as a novel diagnostic technique for assessing surface aging in high-voltage flexible paper insulation. The analysis of dielectric materials demonstrated that the method can effectively detect and localize aged and eroded regions. Additionally, researchers have pointed out that the correlation of hyperspectral patterns with physical degradation mechanisms should be investigated further.

Asset monitoring is critical in hard-to-reach environments, such as offshore wind farms, where the high cost of inspection is driven by logistical challenges and limited accessibility [125]. The in-field inspection of the wind turbine assets often cannot be acquired due to the complexity or fragility of the equipment associated with common methods like X-ray diffraction (XRD), Raman, Fourier transform infrared spectroscopy (FTIR), electrical impedance spectroscopy (EIS) or ultrasonic testing. Additionally, point-based measurements cover limited areas, potentially underestimating damage by failing to provide a complete spatial assessment of spatially distributed patterns. The studies implemented SWIR-HIS FX17 (Specim, Spectral Imaging Ltd., Oulu, Finland), VNIR-HIS SnapScan (IMEC, Leuven, Belgium), SWIR-MSI Bobcat 640 (Xenics NV, Leuven, Belgium), and VNIR-MSI MV0-D2048x1088-C01-HS02-160-G2 (Photonfocus AG, Lachen, Switzerland) cameras to investigate corrosion and coating runoff classification. The sensors have effectively distinguished key corrosion products, surface degradation, and coating defects. Also, the study covered the analysis of the coating quality, the influence of different mixing rations, and the uniformity of the layer (thickness, presence of bubbles, etc.). The study [126] examined another part of the wind turbines—the blades. The tests proved the suitability of spectral imaging for the identification of scratches and erosion of blade specimens. For that purpose, hyperspectral images of the glass-fiber-reinforced polymer blade sample were acquired in a wavelength range of 340–1700 nm. Similar to the application in mines, moisture and dust were considered. The differences in the signatures were reported in the 300–600 nm and 1000–1700 nm ranges. The results of both studies demonstrate the potential for enabling the transition from periodic to condition-based infrastructure maintenance approaches.

## 6. Integration

Recent research on the application of HSI onboard mobile platforms has identified several important integration aspects that are critical for successful deployment in real-world environments. Unlike static or aerial platforms, ground-based mobile systems face unique challenges due to continuous motion, varying terrain, and environmental conditions. These challenges necessitate platform-specific solutions that ensure consistent and reliable spectral data capture during autonomous operation. Key considerations include the following:

- Managing variable illumination;
- Ensuring weatherproofing and environmental protection;
- Adapting scene acquisition to platform and task-specific requirements;
- Handling data large volumes.

### 6.1. Illumination

The application of spectral imaging in outdoor autonomous systems requires consideration of multiple environmental aspects, including the weather conditions, time of day, and surrounding environment (biome/environment). Those variables influence dependent factors like the intensity and spectral distribution of the light, objects present within the scene, and presence of water. Various combinations of these factors not only create unique samples for the analysis but also help to imagine under what conditions a platform equipped with HSI could potentially operate. This issue was addressed by several studies discussing the application of HSI with mobile platforms [51,99,127].

#### 6.1.1. Outdoor Conditions

In nature, the light reaching the ground surface consists of multiple sources. While direct sunlight is one component, there is also light that has been scattered by the atmosphere (known as skylight) and light reflected from various objects [128]. The parameters of those objects, including geometry and material, correlate with the biome. These lighting conditions present unique challenges for ground-based vehicle applications, as both light intensity and spectral characteristics vary dynamically. Understanding these variations is crucial for accurate material classification and scene interpretation. Weather conditions directly affect the quality of the illuminations, as the presence of clouds or direct sun creates different scene properties. It is important to note that urban environments encompass a sophisticated lighting landscape characterized by diverse artificial illumination sources [69,129]. Those light sources might include street lighting, car headlights, advertisement boards, facade lighting, or traffic lights, each with different spectral characteristics. Consequently, objects within a scene are often simultaneously illuminated by multiple light sources, each with unique spectral distributions, which can complicate the process of classifying materials. Four primary approaches to address illumination inconsistencies are as follows:

- White reference calibration;
- Design of the calibration setup;
- Manual exposure time adjustment;
- Active illumination.

The white reference calibration is a common technique used to handle illumination changes in hyperspectral imaging. However, in most of the application cases, the calibration is typically performed prior to acquisition. This approach assumes that the illumination stays consistent throughout the whole acquisition process. To mitigate this assumption, the researchers in [50] equipped the mobile platform with a calibration system. The system utilized two Pebble (Ibsen Photonics, Farum, Denmark) point spectrometers installed inside the weather-sealed housing connected to the external environment via low-OH (Hydroxl) fiber-optic cables (Thorlabs Inc., Newton, NJ, USA). The fiber ends were positioned above a Spectralon (Labsphere, Inc., North Sutton, NH, USA) white reference tile located at the front of the mobile robot. This configuration enabled continuous measurement of the ambient solar spectrum across the 500–1700 nm range. Similarly, in [49], a point spectrometer was used to acquire ground truth illumination in the 225–1000 nm range, utilizing an optical-fiber element.

The next technique to address illumination-related challenges is adjusting the exposure time, which helps improve the quality of the acquired data. In high-intensity lighting, shorter exposure times are effective, while darker environments require more prolonged exposures. Examples of the influence of exposure variations in autonomous driving scenarios are well illustrated in [49]. Similarly, the selection of exposure times is discussed by [67]. However, the situation becomes significantly more complex when both well-illuminated and poorly illuminated areas exist within the same scene, challenging classification algorithms. Such scenarios demand sensors with higher dynamic range capabilities, as indicated in [51]. Currently, to overcome this limitation, researchers typically tune the exposure type of sensors to prevent under- or overexposure or employ per-pixel normalization techniques as a pre-processing step [51]. In low-light conditions, longer exposure times are essential to capture adequate illumination; this creates a critical trade-off by introducing motion blur. Motion blur distorts the appearance of moving objects, such as vehicles and pedestrians. Extended acquisition times may result in the same objects appearing in multiple parts of the scene, leading to spectral mixing in those regions, further complicating image analysis and interpretation.

The challenges associated with varying illumination across a scene were also addressed in [127], where the applicability of the spectral cameras for automatic shadow detection in autonomous driving was investigated in the NIR range. The study aimed to differentiate the regions in the shadows, ignoring dark objects. The results demonstrated that hyperspectral imaging enables the reliable identification of shadowed areas, highlighting its potential for improving scene interpretation under complex lighting conditions.

While the majority of outdoor solutions rely on natural illumination, the VAST sensor setup designed in [66] provided active illumination. Mounted on a Jackal UGV platform (Clearpath Robotics, Kitchener, ON, Canada), this system incorporated a QTH light bulb (Thorlabs Inc., Newton, NJ, USA), which provided consistent illumination of the terrain near each wheel to mitigate temporal lighting variations. To effectively disperse the heat coming from light bulb, the bulb socket was covered with the thermally conductive tape. The researchers used a single onboard spectrometer to gather spectral data from each of four wheels. This was achieved by integrating a micromachined electro-mechanical system (MEMS) fiber-optic switch, which dynamically connected each of the four fiber-optic cables from the wheels to the spectrometer in sequence. So, the data from four wheels were recorded using a single spectrometer.

#### 6.1.2. Indoor Conditions

**Figure 15:**
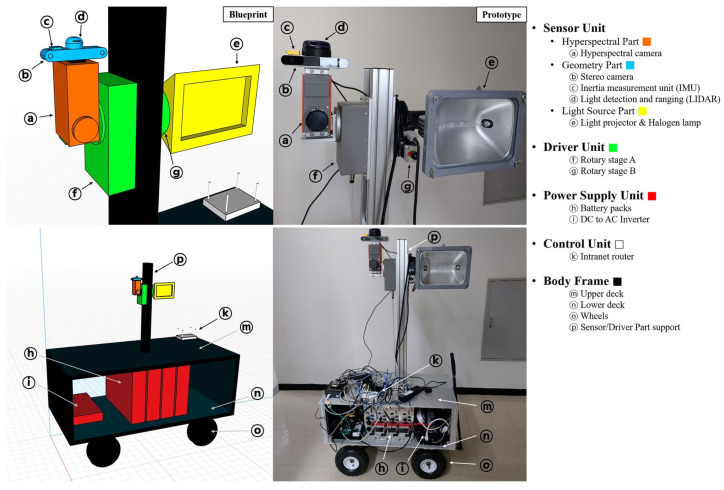
Blueprint and protype of the tunnel coaxial 3D hyperspectral scanning system designed in [65].

To acquire data in indoor environments, platforms must be equipped with active illumination. For example, solutions used in underground mines have employed halogen lighting to illuminate inspection areas [65,67]. In [65], the researchers implemented a 1000 W 189 mm J-type linear halogen lamp with a reflector, as shown in Figure 15. Combined with other system components, the total power consumption reached approximately 1200 W. In [67], three halogen lamps rated at 500 W each were used, resulting in a total power consumption of 1500 W—primarily due to the need to illuminate a wider area for the snapshot sensor. These high-power requirements imply that HSI systems on mobile platforms using halogen lighting must have an extended battery capacity or external power supply.

Another integration-related aspect in [65] is that the HSI pushbroom camera and the halogen light source were mounted on separate rotary stages. This setup was intended to prevent a decrease in the signal-to-noise ratio (SNR) of the sensor caused by heat emitted from the halogen projector. In both cases, white reference calibration was applied to normalize the captured hyperspectral data and compensate for the artificial illumination conditions.

### 6.2. Weather Protection and Optical System Contamination

Several of the previously discussed HSI systems were specifically adapted for operation in challenging weather conditions. In [50,59], the researchers placed the sensors inside epoxy-sealed, weatherproof enclosures. To protect the sensors while minimizing light distortion, an uncoated, chemical-resistant Gorilla Glass (Corning Incorporated, Corning, NY, USA) shielding window was used. Additionally, the FireflEYE Q285 (Cubert GmbH, Ulm, Germany) used in [99] featured a waterproof housing design tailored for outdoor applications.

When considering environmental influences on measurements, it is crucial to account for potential interference from water and foreign materials accumulating on the lens surface. For instance, ref. [51] demonstrated that water droplets on the lens surface substantially degraded segmentation performance. Similar degradation can occur through various environmental mechanisms, including condensation forming directly on optical surfaces and dust accumulation when operating in arid environments. These environmental contaminants can distort the incoming light, reducing system accuracy. Moreover, in [65], the researchers highlighted that the results of scene exploration can be affected by the presence of groundwater vapor and mining dust during the radiometric calibration.

### 6.3. Platform-Specific Scene Acquisition Approaches

When hyperspectral sensors are used for navigation, the scene in front of the platform is typically captured using a snapshot camera. This approach has been applied in several studies [51,59,83,99]. The system must capture scenes at a high frame rate and with sufficient spatial resolution to ensure real-time operation and situational awareness. Although the acquisition time criterion is relevant for the inspection applications as well, it is not associated with the safety risks. This allows for slower acquisition methods, such as scanning, which can provide higher spectral resolution. A representative example is the coaxial 3D hyperspectral scanning system described in [65]. To increase the spatial resolution of the data, two rotary stages for both the light source and pushbroom camera are used.

A similar scanning approach was employed using the Spot quadruped robot [67], which operated in two platform-specific scanning modes. In the first mode, the same scene was acquired using four different vertical pitch angles of the Spot robot, followed by moving along vertical axis to the next position. In the second mode, Spot acquired the sample from the larger distance at three horizontal angles by twisting its body. In both cases, the resulting hyperspectral data segments were merged into a larger “hypercloud”, effectively creating a detailed composite representation of the full scene.

### 6.4. Constraints Defined by the Size of Data

#### 6.4.1. Data Storage

The size of the data is another HSI-specific factor that should be considered while the acquisition. This is the main reason why, in some cases, the throughput of HSI sensors is limited to reduce the memory consumption [92]. As it is stated in [50], the hyperspectral datacubes generated by snapshot cameras have the size of ≈20 megabytes. Twenty-four bands from VNIR sensor and nine bands from the short-wave infrared SWIR sensor contributed to the 33 spectral bands of the hypercube, which has dimensions of 1012 × 1666 × 33. At maximum throughput, the system generates close to 1 GB of data per second, so for the data storage, the three terabytes of solid-state storage were used.

#### 6.4.2. Embedded Processing

The high dimensionality of hyperspectral data introduces a significant bottleneck during both preprocessing and inference stages, particularly when operating on embedded systems. As highlighted in [47], the preprocessing pipeline for raw HSI frames, captured using a snapshot camera with mosaic Fabry–Pérot filters, includes multiple steps such as scene cropping, clipping, reflectance correction, band hypercube formation, and translation to the center.

Due to the limited computational capabilities of embedded devices, on-device scene classification differs considerably from offline processing. To address these limitations, the models used for inference must be lightweight and optimized to balance segmentation accuracy with computational complexity. This involves reducing the number of floating-point operations (FLOPS) and minimizing the memory footprint by lowering the number of parameters [47].

In the study, two key metrics were used to evaluate the computational efficiency of the models: the total number of parameters (NP) and the number of FLOPS. These indicators provide meaningful insight into the resources required for both inference and memory usage. For instance, the U-Net and baseline ANN models exhibited a 3.879-fold difference in FLOPS and a 2.3-fold difference in parameter count, emphasizing the higher computational and memory demands of the U-Net model [47].

To assess real-time performance, the system was tested on three embedded platforms: Raspberry Pi 4B with 8 GB RAM (Raspberry Pi Ltd., Cambridge, UK), Nvidia Jetson Nano (NVIDIA Corporation, Santa Clara, CA, USA) with 4 GB RAM, and the Xilinx ZCU104 FPGA-based development board (AMD, San Jose, CA, USA). The evaluation considered system latency, power consumption, and energy efficiency during both the hypercube preprocessing and inference stages. Power consumption during inference was measured using a USB-C digital meter and dedicated software tools [47].

The fastest mean preprocessing execution time was approximately 24.6 milliseconds on the Jetson Nano board, followed by 51.0 milliseconds on ZCU104 and 52.3 milliseconds on Raspberry Pi. The deployment of the classification model on embedded devices required various platform-specific optimizations, such as thread-level parallelism, SIMD instructions, and the use of dedicated frameworks. During inference, Zynq Ultrascale FPGA consumed 8.8 W, while Raspberry Pi and Jetson Nano consumed 4.258 W and 3.995 W, respectively. In terms of latency, FPGA-based ZCU104 achieved the lowest value at 0.037 s, followed by Jetson Nano at 0.093 s and Raspberry Pi 4B at 0.613 s [47].

The FPGA-based solution delivered nearly 20 frames per second, with the image preprocessing stage identified as the main bottleneck. These results indicate the potential applicability of the system in real-time ADAS scenarios. Further optimization aspects of FPGA-based deployment were discussed in recent publications [130,131].

## 7. Discussion

Integrating HSI in mobile platforms for navigation and inspection tasks represents a complex trade-off between enhanced material perception capabilities and implementation challenges, which are summarized in Table 3 as a SWOT analysis. A key advantage of HSI is its non-invasive nature combined with information-rich sensing capabilities. These sensors help overcome the limitations typical of conventional RGB imaging, such as metamerism and visual similarity issues. Access to detailed material information adds valuable input parameters for mobile platform navigation and provides new capabilities for material-informed inspection.

However, HSI technology has several notable limitations. Being primarily a surface inspection method, HSI may provide insufficient information for decision making in multilayered structures. Environmental factors significantly impact measurement accuracy, including seasonal changes, lighting conditions, and humidity levels. The spectral signatures of materials in a scene are influenced by their intrinsic properties, but the composition of materials also varies with geographic location and seasonal changes. HSI sensors that provide both high spectral and spatial resolution suitable for mobile platform integration typically come with substantial costs. Additionally, these systems show considerable sensitivity to environmental conditions, particularly moisture and dust.

Nevertheless, the current limitations of HSI systems in mobile platforms create opportunities for improvement and more detailed research across various environments and configurations. Different hardware configurations can be evaluated alongside state-of-the-art artificial intelligence methods in mobile scenarios. Furthermore, the optimization of processing algorithms for embedded systems and real-time applications presents an important research direction. New application domains requiring detailed investigation are emerging at the intersection with various novel platforms, particularly in the field of quadruped robotics.

Several risk factors must be considered when looking at future technological developments. Regarding HSI applications beyond the VNIR spectrum, system designs require specific metals such as In, Ga, and Tellurium. The scarcity of these resources could increase manufacturing costs, potentially making these sensors less commercially viable. Additionally, the evolution of technology may be influenced by advances in alternative sensing technologies, such as synthetic aperture radar or advanced RGB image processing methods.

## 8. Conclusions

HSI systems have emerged as powerful tools that enhance mobile platform inspection and navigation capabilities by enabling material-aware perception of the environment. This technology bridges a critical gap in environmental sensing by providing detailed material composition information that conventional sensors cannot detect.

In recent years, significant advancements in HSI integration across diverse mobile platform types, from tracked UGVs and quadruped robots to small, wheeled inspection platforms and conventional vehicles, have been seen, facilitating the investigation of versatile environments. The research performed by several research groups has clearly indicated the potential applicability of HSI for autonomous navigation. The systems were tested in various relevant environments, including public roads, unstructured terrain, and rural environments. Studies have shown that HSI can identify spectral features associated with road surfaces, vegetation, and obstacles. Moreover, recent research involving embedded platforms, including FPGA-based systems, indicated the feasibility of deploying HSI in real-world scenarios. Another relevant output of those studies is several datasets, including ADAS-relevant scenes.

In inspection applications, HSI enables the non-destructive evaluation of material conditions, detection of degradation patterns, and early identification of potential failures. This material-aware inspection approach significantly advances traditional visual inspection methods, offering quantitative data for condition assessment and maintenance planning. Platforms equipped with HSI can provide assistance in mining applications or during search and rescue operations.

Despite its potential, successful HSI integration faces several key challenges that require further development. Integration with multi-sensor systems demands robust fusion strategies and platform-specific optimizations to manage power consumption, processing resources, and physical constraints. For mobile platforms, these challenges are amplified by motion-induced artifacts, vibration effects, and the need for real-time processing capabilities. Environmental factors significantly impact HSI performance, with measurement quality varying based on the weather conditions, time of day, and local environmental characteristics. Understanding these environmental dependencies and platform-specific limitations is crucial for defining operational parameters and developing compensation strategies for reliable deployment across different scenarios. The integration complexity varies with platform type—from the high-vibration environments of tracked vehicles to the dynamic motion patterns of quadruped robots—requiring tailored mounting, stabilization, and data acquisition solutions.

As HSI technology applied with mobile platforms continues to mature, further research focusing on environmental robustness, real-time processing optimization, and the standardization of data acquisition methods will be crucial for establishing it as a standard sensing modality in mobile robotics applications. With continued development, HSI has the potential to become an integral component of conventional sensor suites, enhancing the material-awareness capabilities of mobile platforms.

## Figures and Tables

**Table 1:** Applicability of HSI acquisition modes for navigation and inspection.

Acquisition Mode	Point Scanning	Line Scanning	Area Scanning	Snapshot
Sensitivity to moving and vibration	High	High	High	Low
Spectral resolution	High	High	Low	Moderate
FOV per measurement	Low	Moderate	High	High
Acquisition speed	Low	Low	High	Low
Dependence of spatial resolution on acquisition speed	High	Moderate	Moderate	Low
Spatial resolution is dependent on platform motion or internal scanning components	Yes	Yes	No	No
Applicability for inspection	High	High	Moderate	Moderate
Applicability for navigation	Low	Moderate	Low	High

**Table 3:** SWOT analysis of HSI application for navigation and inspection on mobile robots.

Strengths	Weaknesses
Metamerism tolerant Information-rich source Passive sensing technology Non-destructive and non-contact	Sensitivity to illumination Sensitivity to dust/moisture on the optics Location dependent Intraclass variability influence High sensor cost By default, the imbalanced nature of the datasets Surface inspection method
**Opportunities**	**Threats**
Real-time HSI classification Investigation of new environments (geography) Automated acquisition parameter tuning New sensor design Fusion with other sensors Integration of state-of-the-art algorithms Optimization for embedded systems Utilization of spatiotemporal approaches Integration with new platforms (incl. quadruped robots)	Data storage Advances in the design of other sensors and data processing Competition from alternative sensing technologies Supply chain issues affecting rare materials Slow adoption in industry due to high initial costs and technical complexity

## Abbreviations

The following abbreviations are used in this manuscript:

2D	Two dimensional
3D	Three dimensional
AGAS	Active Vision Group
ACI	Asphalt Crack Index
ADAS	Advanced Driver Assistance System
ANN	Artificial Neural Network
AOTF	Acousto-Optical Tunable Filters
CCD	Charge-Coupled Device
CMOS	Complementary Metal-Oxide Semiconductor
DN	Digital Number
DVE	Degraded Visual Environment
EIS	Electrical Impedance Spectroscopy
FCN	Fully Convolutional Network
FPGA	Field Programmable Gate Array
FOV	Field of View
FTIR	Fourier Transform Infrared Spectroscopy
FCLS	Fully Constrained Least Squares
Ga	Gallium
GIFOV	Ground Instantaneous Field of View
GSD	Ground Sample Distance
HSI	Hyperspectral Imaging
IFOV	Instantaneous Field of View
IMU	Inertial Measurement Unit
In	Indium
HgCdTe	Mercury Cadmium Telluride
InGaAs	Indium Gallium Arsenide
InSb	Indium Antimonide
IR	Infrared
JPL	Jet Propulsion Laboratory
LCTF	Liquid Crystal Tunable Filter
LED	Light-Emitting Diode
LiDAR	Light Detection and Ranging
LWIR	Long-Wave Infrared
MNF	Minimum Noise Fraction
MSI	Multispectral Imaging
MWIR	Mid-Wave Infrared
NASA	National Aeronautics and Space Administration
NIR	Near-Infrared
PCA	Principal Component Analysis
PCI	Pavement Condition Index
PLSDA	Partial Least Squares Discriminant Analysis
RGB	Red–Green–Blue
Si	Silicon
Si:As	Silicon doped with Arsenic
SNR	Signal-to-Noise Ratio
SWIR	Short-Wave Infrared
SVM	Support Vector Machine
Te	Tellurium
UAV	Unmanned Aerial Vehicle
UGV	Unmanned Ground Vehicle
UV	Ultraviolet
VIS	Visible Light
VNIR	Visible and Near-Infrared
XRD	X-ray Diffraction
